# Urinary proteome signature of Renal Cysts and Diabetes syndrome in children

**DOI:** 10.1038/s41598-019-38713-5

**Published:** 2019-02-18

**Authors:** Pierbruno Ricci, Pedro Magalhães, Magdalena Krochmal, Martin Pejchinovski, Erica Daina, Maria Rosa Caruso, Laura Goea, Iwona Belczacka, Giuseppe Remuzzi, Muriel Umbhauer, Jens Drube, Lars Pape, Harald Mischak, Stéphane Decramer, Franz Schaefer, Joost P. Schanstra, Silvia Cereghini, Petra Zürbig

**Affiliations:** 1 Sorbonne Université - CNRS - UMR7622 - Institut de Biologie Paris Seine (IBPS), Paris, France; 2 Mosaiques Diagnostics GmbH, Hannover, Germany; 3 Department of Pediatric Nephrology, Hannover Medical School, Hannover, Germany; 4 IRCCS – Istituto di Ricerche Farmacologiche Mario Negri – Clinical Research Center for Rare Diseases Aldo e Cele Daccò, Ranica Bergamo, Italy; 5 Unit of Nephrology, ASST Papa Giovanni XXIII, Bergamo, Italy; 6 University Hospital RWTH Aachen, Institute for Molecular Cardiovascular Research (IMCAR), Aachen, Germany; 7 Service de Néphrologie Pédiatrique, Hôpital des Enfants, CHU Toulouse, Toulouse, France; 8 Centre De Référence des Maladies Rénales Rares du Sud Ouest (SORARE), Toulouse, France; 9 Institut National de la Santé et de la Recherche Médicale (INSERM), U1048, Institut of Cardiovascular and Metabolic Disease, Toulouse, France; 10 Université Toulouse III Paul-Sabatier, Toulouse, France; 11 University Children Hospital, Pediatric Nephrology, Heidelberg, Germany

## Abstract

Renal Cysts and Diabetes Syndrome (RCAD) is an autosomal dominant disorder caused by mutations in the *HNF1B* gene encoding for the transcriptional factor hepatocyte nuclear factor-1B. RCAD is characterized as a multi-organ disease, with a broad spectrum of symptoms including kidney abnormalities (renal cysts, renal hypodysplasia, single kidney, horseshoe kidneys, hydronephrosis), early-onset diabetes mellitus, abnormal liver function, pancreatic hypoplasia and genital tract malformations. In the present study, using capillary electrophoresis coupled to mass spectrometry (CE-MS), we investigated the urinary proteome of a pediatric cohort of RCAD patients and different controls to identify peptide biomarkers and obtain further insights into the pathophysiology of this disorder. As a result, 146 peptides were found to be associated with RCAD in 22 pediatric patients when compared to 22 healthy age-matched controls. A classifier based on these peptides was generated and further tested on an independent cohort, clearly discriminating RCAD patients from different groups of controls. This study demonstrates that the urinary proteome of pediatric RCAD patients differs from autosomal dominant polycystic kidney disease (*PKD1*, *PKD*2), congenital nephrotic syndrome (*NPHS1*, *NPHS2*, *NPHS4*, *NPHS9*) as well as from chronic kidney disease conditions, suggesting differences between the pathophysiology behind these disorders.

## Introduction

Renal Cysts and Diabetes (RCAD) syndrome is caused by heterozygous mutations in the *HNF1B* gene, encoding the transcriptional factor hepatocyte nuclear factor-1B. RCAD syndrome (RCAD, OMIM #137920)^1^ can also be referred as MODY5 (Maturity Onset Diabetes of the Young type 5)^2^. The wide spectrum of clinical features in RCAD patients is due to the multisystem role of HNF1B, which is involved in normal morphogenesis of several organs, including kidneys, pancreas, liver, and genitourinary tract^3^. Consistent with its broad developmental expression pattern^4,5^, studies on fetuses carrying *HNF1B* mutations revealed a fundamental function during kidney, urogenital tract and pancreas development^4,6–8^.

The RCAD disease is inherited in an autosomal dominant pattern^9^. To date, more than 150 mutations have been described in the *HNF1B* gene. Half of the RCAD patients characterized up to date present missenses, nonsenses, frameshifts, splice site mutations, and insertions/deletions while the other half of patients present whole gene deletions^10^.

The most prominent clinical feature in *HNF1B*-associated syndrome is the renal disease, usually characterized by renal cysts, renal dysplasia, solitary or horseshoe kidney, hydronephrosis, and hyperuricaemic nephropathy^11^. The renal abnormalities of *HNF1B* mutant carriers have also been related to the congenital anomalies of the kidney and the urinary tract (CAKUT)^11^. Moreover, recently, *HNF1B*-mutations have been associated in some patients to autosomal dominant tubulointerstitial kidney disease (ADTKD-HNF1B)^12^. In addition, several cases of unknown chronic kidney disease (CKD) have been reported both in children^13^ and adults^14,15^. Tubular dysfunction manifesting by hypomagnesemia, hypocalciuria^16–18^, and hyperuricemia^14,19^ has also been described. Extrarenal features comprise maturity-onset diabetes of the young, pancreatic hypoplasia, abnormal liver function, and genital tract malformations. The phenotype of *HNF1B* mutant carriers is indeed highly variable within and between families^20^. These observations led to the hypothesis that non-allelic factors, as well as stochastic variation in temporal *HNF1B* gene expression and environmental factors, could cause the strong intrafamilial variability of RCAD patients^3,21^.

Urinary proteomics is increasingly being employed in kidney disease research. Several studies have demonstrated that capillary electrophoresis coupled to mass spectrometry (CE-MS) enables the identification and validation of several biomarkers or peptide signatures classifying the diagnosis and prognosis of various kidney diseases^22–25^. In addition to their diagnostic and prognostic usefulness, proteomics derived biomarkers may advance the understanding of the molecular pathways involved in the pathogenesis of a specific disorder or condition.

In this study, we aimed to obtain more insights into the renal pathophysiology of the RCAD syndrome by applying a proteomic approach to investigate changes at urinary peptides level that can be used to characterize RCAD patients.

## Results

### Study setup and patient data

**Figure 1:**
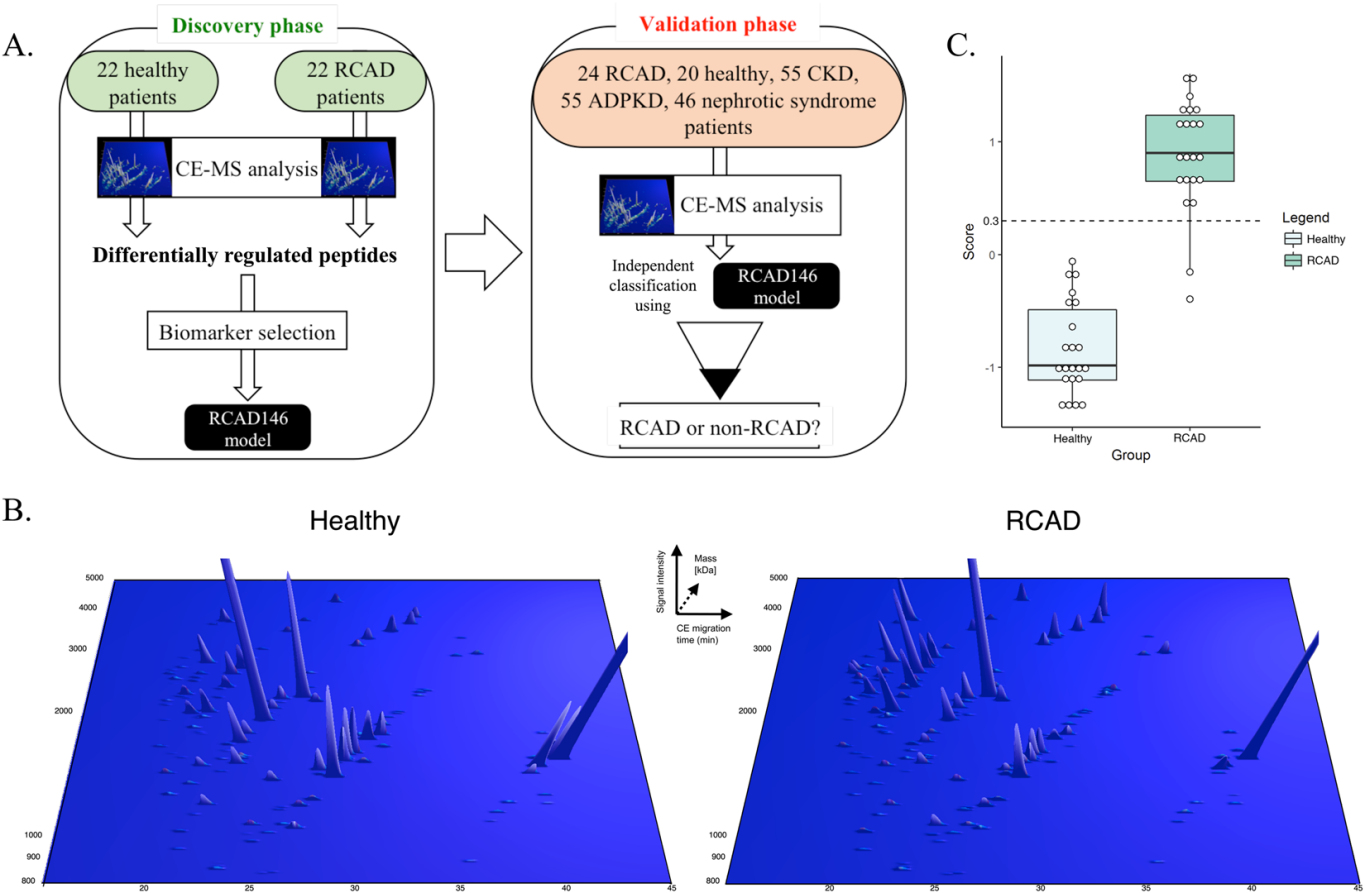
Study design and urinary CE-MS analysis of patients with RCAD. (**A**) The analysis was performed in two phases: a discovery phase, where the urinary proteome of 44 pediatric patients (22 healthy, 22 RCAD) was analyzed leading to the identification of 146 sequenced urinary peptides that were modelled in a SVM classifier called RCAD146. In the next step, the validation phase, we studied the discriminatory ability of the panel RCAD146 panel in new RCAD patients (*n* = 24) and individuals with CKD or patients carrying monogenic mutations associated with different renal diseases. (**B**) Representation of the 146 urinary peptides significantly modified between RCAD and healthy controls. Normalized molecular mass (kDa) was plotted against normalized capillary electrophoresis (CE)-migration time (min). Mean signal intensity was given in 3-dimensional depiction. (**C**) Cross-validation score of the RCAD146 model from the analysis of the discovery cohort along with the definition of the cut-off 0.3 (dashed line).

In total, 244 urine samples were included in this study: 44 samples were used for discovery and 200 samples were used as a validation set (Fig. 1A). In the discovery set, we included 22 RCAD patients and 22 healthy controls. Subsequently, we used a wider population comprising healthy patients (*n* = 20), RCAD patients (*n* = 24), autosomal dominant polycystic kidney disease (ADPKD) patients (*n* = 55), CKD patients (*n* = 55), and patients with nephrotic syndrome (*n* = 46) as a validation set. The urinary proteome data for all the samples were previously measured and derived from the Human Urinary Proteome Database^26–28^ with the exception of all RCAD urine samples, which were analyzed by CE-MS for this specific study. The 46 RCAD patients were divided into discovery and validation set. The RCAD samples used in the discovery were matched with healthy controls based on age and gender. Furthermore, we divided both sets considering similar phenotypes. The clinical features of the used RCAD patients are described in Table 1 and Supplementary Table 1.

**Table 1:** Clinical characteristics of children with RCAD syndrome.

	All
***n***	46
**Male**, **n (%)**	37 (80.4)
**Female**, **n (%)**	9 (19.6)
**Age**, **years**	8.4 ± 5.7
**eGFR (ml/min/1**.**73 m**^**2**^**)**	86.74 ± 41.15
**U-protein (g/L)**	0.12 ± 0.35
**Renal phenotypes**	
kidney cysts, *n* (%)	34 (73.9)
bilateral hyperechoic kidneys, *n* (%)	24 (52.1)
hypo/dysplastic kidneys, *n* (%)	21 (45.6)
single kidneys, *n* (%)	3 (6.5)
vesicoureteral reflux, *n* (%)	2 (4.3)
horseshoe kidneys, *n* (%)	1 (2.1)
chronic renal failure, *n* (%)	1 (2.1)
**Extrarenal phenotypes**	
diabetes, *n* (%)	3 (6.5)
pancreatic hypoplasia, *n* (%)	3 (6.5)
uterine malformations, *n* (%)	2 (4.3)
unilateral ectopic testis, *n* (%)	2 (4.3)
hyperechoic liver, *n* (%)	1 (2.1)
cholestasis, *n* (%)	1 (2.1)
megabladder, *n* (%)	1 (2.1)
hyperuricemia, *n* (%)	5 (10.8)

### Identification of RCAD-related urinary peptides and development of a urinary peptide-based classifier

For the identification of significant urinary peptides related to the RCAD syndrome, we compared the urinary proteome profiles of 22 patients carrying *HNF1B* heterozygous mutations with 22 age- and gender-matched healthy controls (Table 2A). The statistical analysis was adjusted for multiple testing following the concept described by Benjamini and Hochberg^29^ and defined in the clinical proteomics guidelines^30^. This led to the identification of 294 differentially excreted peptides (corrected p < 0.05) between these two groups. For 146 out of the 294, high-confidence sequence information could be assigned. Fragments of uromodulin (UMOD), protein unc-119 homolog A (UNC119), and mucin (MUC1), as well as a large number of collagen fragments, were identified. Moreover, peptides associated with calcium binding were also detected. Amongst them, peptides such as sarcalumenin (SRL), and annexin A1 (ANXA1) were downregulated. In contrast, peptide fragments such as gelsolin (GSN), short transient receptor potential channel 4-associated protein (TRPC4AP) and the direct target of HNF1B - osteopontin (SPP1)^31^ were upregulated. All relevant details, encompassing the sequence information as well as the fold-change, are described in Supplementary Table 2. The difference in abundance of these 146 peptides between RCAD patients and healthy controls is shown in Fig. 1B and Table 3. These proteome plots are showing the mean abundance of the significant peptide-biomarker in urine of RCAD patients and healthy individuals. The 146 sequenced peptides were combined into a classifier termed “RCAD146” using a support vector machine (SVM), which was optimized for the classification of patients in the discovery cohort. Based on a cut-off score of 0.30 (Fig. 1C), the RCAD classifier discriminated RCAD from healthy controls with 90.9% sensitivity and 100% specificity and an AUC of 0.99 in the discovery cohort.

**Table 2:** Baseline characteristics of the subjects used in the A. discovery set and B. validation set.

Group of patients	Mutation	Sample size *(n)*	Gender		Age (years)	eGFR (ml/min/1.73 m^2^)	U-albumin (mg/l)	U-protein (g/l)
			Male	Female				
**A**. **Discovery Set**								
RCAD	HNF1B	22	17	5	9.4 ± 6.4	81.0 ± 43.67		0.18 ± 0.50
Healthy	—	22	17	5	9.3 ± 4.1	126.33 ± 30.03	—	—
**Patients’ condition**	**Mutation**	**Sample size** ***(n)***	**Gender**		**Age (years)**	**Mean eGFR (ml/min/1**.**73 m**^**2**^**)**	**U-albumin (mg/l)**	**U-protein (g/l)**
			**Male**	**Female**				
**B**. **Validation Set**								
RCAD	HNF1B	24	20	4	7.5 ± 5.1	92.03 ± 38.88		0.07 ± 0.08
Healthy	—	20	15	5	9.3 ± 2.4	118.5 ± 38.32	—	—
CKD	—	55	28	27	12.0 ± 6.1	69.79 ± 23.94	867.65 ± 1673.49	—
ADPKD	PKD1	46	20	26	34.3 ± 6.0	77.33 ± 18.08	—	
	PKD2	9	3	6	40.2 ± 3.3			—
Nephrotic Syndrome	NPHS1	2	2	—	6.5 ± 8.8	108.50 ± 80.95	244.14 ± 398.23	—
	NPHS2	35	14	21	10.5 ± 6.4			
	NPHS4 (WT1)	6	1	5	13.3 ± 3.7			
	NPHS9 (ADCK4)	3	1	2	13.6 ± 3.1

**Table 3:** Proteins origin of the 146 differentially excreted urinary peptides obtained by the comparison between RCAD and healthy patients.

Protein name	Gene symbol	N° of protein fragments	P-value (adjusted)	Mean fold change
Mucin-3a	MUC3A	1	1.10E-05	−56.9
Collagen alpha-1 (XXVI) chain	COL26A1	1	1.72E-03	−11.2
Protein unc-119 homolog A	UNC119	1	4.95E-03	−7.05
Collagen alpha-3 (V) chain	COL5A3	1	7.70E-04	−6.25
Uromodulin	UMOD	4	4.82E-02	−5.7 (±2.33)
Collagen alpha-1 (XXVII) chain	COL27A1	1	2.04E-02	−5.66
Cystatin-A	CSTA	1	5.88E-03	−3.67
Hemoglobin subunit delta	HBD	1	3.65E-02	−3.38
Annexin A1	ANXA1	1	6.44E-03	−2.68
Sarcalumenin	SRL	1	1.05E-02	−2.21
Collagen alpha-1 (XVII) chain	COL17A1	1	3.14E-03	−2.03
Ig lambda-2 chain C regions	IGLC2	1	1.45E-02	−1.92
Beta-2-microglobulin	B2M	1	1.04E-02	−1.17
Serum amyloid A protein	SAA1	2	4.87E-02	−0.81 (±2.95)
Collagen alpha-1 (XI) chain	COL11A1	1	4.21E-03	1.19
Collagen alpha-1 (VIII) chain	COL8A1	1	3.61E-02	1.29
Kininogen-1	KNG1	1	1.69E-02	1.53
Collagen alpha-1 (I) chain	COL1A1	52	4.97E-02	1.7 (±3.42)
Short transient receptor potential channel 4-associated protein	TRPC4AP	1	1.74E-02	1.98
Collagen alpha-2 (V) chain	COL5A2	3	1.67E-02	2.23 (±2.9)
Collagen alpha-1 (XVI) chain	COL16A1	1	3.53E-02	2.24
Mucin-1 subunit alpha	MUC1	1	9.90E-03	2.4
Ig kappa chain C region	IGKC	1	1.68E-02	2.64
Collagen alpha-5 (IV) chain	COL4A5	1	2.88E-02	2.86
Collagen alpha-6 (IV) chain	COL4A6	1	5.61E-04	3
Retinol binding protein 4	RBP4	1	7.78E-03	3.11
Protein scribble homolog	SCRIB	1	3.51E-02	3.15
Collagen alpha-2 (I) chain	COL1A2	20	4.84E-02	3.28 (±6.86)
Actin, cytoplasmic 1	ACTB	1	2.88E-02	3.3
Neurosecretory protein VGF	VGF	1	3.80E-02	3.45
Collagen alpha-1 (III) chain	COL3A1	21	4.67E-02	3.47 (±4.54)
Osteopontin	OPN	2	1.56E-02	3.85 (±2.21)
Collagen alpha-4 (IV) chain	COL4A4	1	1.46E-03	4
Collagen alpha-1 (II) chain	COL2A1	7	3.05E-02	4.45 (±5.37)
Fibrinogen alpha chain	FGA	2	5.87E-03	4.79 (±0.48)
Ephrin-A1	EFNA1	1	5.46E-03	5.55
Interleukin-1 receptor-associated	IL-1R	1	1.27E-04	43.2
Immunoglobulin kappa variable 4-1	IGKV4-1	1	1.88E-04	56.7
Gelsolin	GSN	1	1.66E-05	105.4
Microfibrillar-associated protein 5	MFAP5	1	4.32E-05	308.3
Collagen alpha-1 (V) chain	COL5A1	2	4.36E-02	180.32 (±253)

Proteins origin of the 146 differentially excreted urinary peptides obtained by the comparison between RCAD and healthy patients. An extended detailed version of the table can be found in Supplementary Table 2. Data described the number of significant peptides related to each protein, the lowest observed P-value and the mean fold change (±standard deviation).

### Validation of the RCAD146 classifier in an independent group

**Figure 2:**
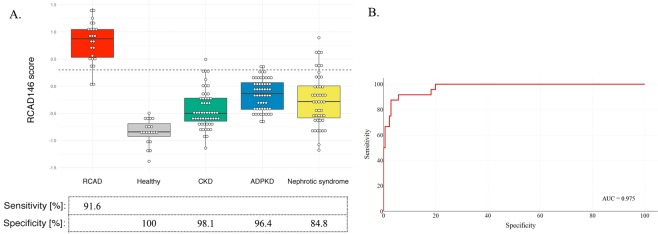
Blinded validation of urinary peptide classifier RCAD146 in a separate RCAD patient population together with healthy controls and patients suffering from other kidney diseases. (**A**) Box-and-Whisker plot for the classification of all patient cohorts (RCAD subjects, non-RCAD groups) of the validation set according to the RCAD146 scores. (**B**) ROC curve for the RCAD146 model based on all samples used in the validation cohort.

The RCAD146 classifier was validated in an independent group of samples (Table 2B), consisting of 24 RCAD and 20 healthy controls. The analysis revealed an AUC of 1.00 [0.92 to 1.00 (95% CI); p < 0.0001]. At the pre-defined cut-off level of 0.30 based on the discovery cohort, the classifier displayed a sensitivity of 91.67% and specificity of 100%. To obtain confirmation about how RCAD146 performed in differentiating the pediatric RCAD urinary proteome from other kidney diseases, we further selected a group of patients with chronic kidney disease (CKD) (*n* = 55). This control group of children was particularly interesting as (i) 40% of adults with HNF1B mutations develop CKD^14^, (ii) it represents a condition with severe chronic kidney damage and, (iii) confirms that the performance of the RCAD146 classifier is independent of proteinuria in RCAD patients. This analysis showed a specificity of 98.18% and an AUC of 0.987 [0.931 to 1.00 (95% CI); p < 0.0001] for the classification of children with CKD as non-RCAD. To further evaluate the specificity and validity of the pediatric RCAD urinary proteomic pattern, the classifier was subsequently tested in patients with different monogenic kidney diseases, including ADPKD and nephrotic syndrome. In the case of ADPKD, 53 out of 55 patients were scored as non-RCAD corresponding to a specificity of 96.4% [AUC: 0.974] (Fig. 2A). In the group of patients with nephrotic syndrome, 39 of 46 patients were scored by RCAD146 as non-RCAD displaying a specificity of 84.8% [AUC: 0.952] (Fig. 2A).

When evaluating all data sets combined, the overall sensitivity and specificity were 91.67% and 94.32%, respectively. Furthermore, this ROC analysis including all datasets revealed an AUC of 0.975 [0.943 to 0.992 (95% CI); p < 0.0001] (Fig. 2B).

## Discussion

This is the first study showing a unique proteome profile that distinguishes children with RCAD from healthy controls and patients suffering from different renal diseases.

The most prominent finding of the study was the identification of 294 differentially regulated peptides potentially related to RCAD syndrome, where sequence information was obtained for 146 peptides. Similar to a previous study on ADPKD and urinary peptides^32^, the majority of peptides enriched in the urine of RCAD patients were collagen type I or type III fragments. This may reflect active extracellular matrix (ECM) remodelling, which could be related to ECM modifications due to cyst expansion^33^. The abundance of collagen and osteopontin fragments in RCAD children displayed an opposite tendency to previous findings described in several studies for different kidney diseases^22,23,34^. Collagens are characterized as the most abundant urinary peptides as well as the main elements of the interstitial ECM, being involved in different biological functions as cell adhesion, tissue development and tensile strength^35,36^. Along these lines, osteopontin-derived peptides were also identified increased in RCAD patients, due to the involvement of osteopontin in the remodelling of the ECM^34^. Therefore, the increased abundance of collagen and osteopontin fragments in the RCAD urinary samples may reflect the cystic phenotype and the still non-fibrotic status of patients’ kidneys, whereas tubulointerstitial fibrosis determines the peptide excretion pattern in CKD^37^. Additionally, an early decline in kidney function may be predicted by the decreased excretion of uromodulin (UMOD) fragments^38^, which has also been found reduced in cases with tubular atrophy and fibrosis^39^. Another peptide fragment deregulated in the present study was mucin 1 (MUC1), an extracellular protein expressed in the renal tubular epithelium. Recently, MUC1 was described to be a predictor of renal impairment^40^, along with its increase in mice and human was correlated with the development of fibrosis^41^. It is important to notice that mutations in UMOD, MUC1, and HNF1B are responsible for ADTKD, showing a strict correlation between these proteins and RCAD along with ADTKD phenotypes^42^. Considering the acknowledged role of HNF1B in regulating kidney transports and also calcium-sensing receptor CaSR^16,43^, it was interesting to notice that several peptides associated with calcium binding or calcium regulating properties might be changed in RCAD patients. The disruption of multiple calcium regulators may be one of the bases of the renal cysts formation as observed previously^44,45^. Furthermore, the protein unc-119 homolog A (UNC119) that plays a crucial role in the proper ciliary targeting of the cystic gene nephrocystin-3^46^, was decreased.

The RCAD146 classifier correctly identified most of CKD patients as non-RCAD. Since CKD is a rare condition in RCAD children^13^, it would be of interest testing a cohort of adult RCAD patients suffering from CKD, in order to investigate the performance of the RCAD146 classifier.

The other disorders used as disease controls in this study were biologically related (e.g. ADPKD) or non-related (e.g. nephrotic syndrome) to the RCAD syndrome. A group of patients affected by ADPKD appears relevant as they display phenotypic correlations with the RCAD syndrome. HNF1B was shown to regulate *Pkd2* in the mouse^47^ and mutations in *HNF1B* can mimic polycystic kidney disease especially in the prenatal setting and early childhood^48,49^. Notably, the RCAD146 classifier precisely discriminated RCAD from ADPKD.

When compared with monogenic mutations sharing a common nephrotic syndrome phenotype, the RCAD146 classifier also identified subjects carrying mutations in *NPHS1*, *NPHS2*, *WT1 (NPHS4)*, and *ADCK4* (*NPHS9*) as non-RCAD. This group of patients may confirm, as the CKD group, that RCAD urinary proteome is not reflecting just proteinuria.

Interestingly, in a parallel test, the urinary proteome of a two year old *PAX2* mutant carrier was misrecognized by RCAD146 (data not shown). This observation suggests that the RCAD pediatric proteome could potentially be closer to patients with mutations in the gene encoding the transcription factor PAX2, known to cooperate with HNF1B in kidney morphogenesis and ureter differentiation^50^, than patients with either polycystic or nephrotic syndrome. Additional samples are required to further validate this common feature.

A limitation of the current study is that the recruited ADPKD patients were not children, but young adults. This is due to the difficulty in recruiting children with ADPKD because the average age at the diagnosis is 30 years old^51^. Another shortcoming is that there was no information available related to the respective albuminuria/proteinuria values of the ADPKD patients. Moreover, this study included a post hoc analysis, due to the selection of the diseased control population from previous studies^37,52–57^. However, all the samples were analysed according to the same rules and identical conditions (sample preparation and proteomic platform). No discrepancy between the data of the measured RCAD samples and the stored data is to be expected, because the normalization procedure protects the data from aberration of the intensity of the peptide signals. Furthermore, we controlled all measurements with a urine standard sample to identify unforeseeable technical aspects over time^58^.

Overall, the study, performed in agreement with the guidelines of clinical proteomics, demonstrates a significant value of the urinary proteome analysis in the detection of RCAD highlighting some proteins that potentially participate in the development of cysts and that may be useful for early diagnosis.

The urinary peptide signature of pediatric RCAD patients is mainly characterized by the increase of collagen peptides (especially type I or type III fragments), and osteopontin, along with the decrease of uromodulin. Including the 146 peptides differentially excreted between RCAD and healthy patients in a diagnostic biomarker classifier, we demonstrated that RCAD pediatric urinary proteome is different from patients with *Pkd1-2* and *Nphs1-2-4-9* mutations, as well as from CKD patients. Future studies will be conducted to evaluate the performance of the RCAD146 panel in additional pediatric cohorts of disorders more related to RCAD such as autosomal recessive polycystic kidney disease (ARPKD), ADTKD or diabetic patients. Moreover, follow-up clinical data of the patients described in this study will be addressed to estimate the performance of this classifier to predict the progression of RCAD. These analyses together are expected to provide further insights into the pathophysiology and disease evolution of RCAD patients.

## Methods

### Patient recruitment

RCAD urine samples were collected from three different clinical centres: Children’s Hospital, CHU-Toulouse (France, *n* = 33), University Children Hospital, Heidelberg (Germany; *n* = 11), Clinical Research Center for Rare Diseases Aldo e Cele Daccò, Ranica (Italy, *n* = 2). RCAD patients’ average age was 8.4 years. Furthermore, 56.5% of the RCAD patients had a normal renal function (estimated glomerular filtration rate (eGFR) > 90 ml/min/1.73 m^2^). For patients under 20 years, baseline eGFR (mL/min/1.73 m2) was estimated using the creatinine-based “Bedside Schwartz” equation^59^. On the other hand, for patients over 20 years (e.g. ADPKD cohort), the CKD-EPI formula was used to calculate the eGFR values^60^. After collection, urine samples were stored at −20 °C and shipped frozen for subsequent proteome analysis. In addition, all non-RCAD samples were retrieved from the Human Urinary Proteome database^26–28^. This group of samples included healthy patients (*n* = 42), and patients suffering from kidney diseases and carrying different genetic mutations, such as: *PKD1* (*n* = 46); *PKD2* (*n* = 9), *NPHS1* (*n* = 2), *NPHS2* (*n* = 35), *WT1* (*n* = 6), *ADCK4* (*n* = 3). Additionally, a group of samples from a pediatric cohort with chronic kidney disease was tested with different etiologies, like focal segmental glomerulosclerosis, IgA nephropathy, membranous glomerulonephritis, mesangioproliferative glomerulonephritis, diabetic nephropathy, vasculitis, and Henoch-Schönlein purpura nephritis (*n* = 55). This wider group of negative controls (non-RCAD) presented an average age of 16.5 years. RCAD and healthy patients were selected by similar age and gender; CKD and nephrotic syndrome cohorts were age-matched and ADPKD patients were phenotypic-matched for the presence of cysts. Characteristics of all individuals included in this study are extended in Supplementary Table 1.

This study was designed and performed in compliance with all the regulations regarding the protection of subjects participating in medical research. Collection, storage and analysis of urine samples have been approved by the local ethics committees of the three participating centres (Comité de Protection des Personnes Sud-Ouest et Outre Mer III, Ethikkommission der Medizinischen Fakultät Heidelberg, and Comitato Etico di Bergamo respectively). All participating subjects or legal guardians of patients provided written informed consent to the use of urine samples. This study was performed in accordance with the Helsinki Declaration.

### Urine sample preparation and CE-MS analysis

Urine sample collection and CE-MS analysis were performed as reported previously^61,62^. Briefly, immediately before preparation, urine samples aliquots stored at −20 °C were thawed and 700 μl were diluted with the same volume of 2 M urea, 10 mM NH_4_OH comprising 0.02% SDS. Then, samples were filtered via a Centristat 20-kDa cut-off centrifugal filter device (Sartorius, Goettingen, Germany) at 2,600 g for one hour at 4 °C in order to remove high molecular weight compounds. The obtained filtrate was desalted using a PD-10 column (GE Healthcare, Sweden) equilibrated in 0.01% aqueous NH_4_OH to eliminate urea, electrolytes and salts. Finally, samples were lyophilized and stored at 4 °C prior to CE-MS analysis. The samples were re-suspended in 10 µL of HPLC-grade H_2_O shortly before CE-MS analysis, as described^62^. CE-MS analyses were accomplished using the P/ACE MDQ capillary electrophoresis system (Beckman Coulter, Fullerton, USA) online coupled to a MicroTOF MS (BrukerDaltonic, Bremen, Germany)^62^. The electro-ionization sprayer (Agilent Technologies) was grounded, and the ion spray interface potential was defined between −4 and −4.5 kV. Spectra were accumulated every 3 s along with over a range of m/z to 350–3000. Detailed information on accuracy, precision, selectivity, sensitivity, reproducibility and stability of the CE-MS method have been described previously^62^.

### CE-MS data processing

A proprietary software (MosaiquesVisu) was used to deconvolute mass spectral ion peaks demonstrating identical molecules at different charge states into single masses^63^. The achieved peak list allows the characterization of each polypeptide according to its CE-migration time (in minutes), molecular mass (in Daltons), and ion signal intensity. Subsequently, normalization of the amplitude of the urinary peptides was conducted on twenty-nine ‘housekeeping’ peptides (peptides varied slightly between samples, generally present in at least 90% of all urine), similarly to previous studies^64^. These 29 ‘housekeeping’ peptides are commonly used for normalization in all studies. Furthermore, these peptides are consistently reported in urine and to date, they do not appear to be significantly associated with any diseases investigated^64^. All detected peptides were deposited, clustered, matched and annotated in a Microsoft SQL database^26–28^, allowing further statistical analysis. All normalized amplitudes of the analysed samples are included in Supplementary Table 3.

### Peptide sequencing

Candidate peptides for the RCAD-classifier were identified and sequenced by the use of tandem mass spectrometry (MS/MS) analysis and searched against human entries in the UniProt database, as previously described^65,66^. Briefly, to acquire the sequence information, urine samples were separated on a Dionex Ultimate 3000 RSLC nano flow system (Dionex, Camberly, UK) or a Beckman CE systems (PACE MDQ) coupled to an Orbitrap Velos MS instrument (Thermo Fisher Scientific)^65,66^. Thereafter, data files were examined against the UniProt human non-redundant database using Proteome Discoverer 1.2 (Thermo) and the SEQUEST search engine. No fixed modifications were selected, hydroxylation of proline and lysine and oxidation of methionine were enabled as an optional modification, no enzyme specificity was specified in the settings^65^. The matching of the peptide sequence obtained by MS/MS analysis to the CE-MS peaks was based on molecular mass [Da] and theoretical migration time, calculated using the number of basic amino acids^67^. Peptides were accepted only if they had a mass deviation below ±5 ppm and <50 mDa for the fragment ions.

### Peptide identification and statistical analysis

For the identification of potential HNF1B-related urinary peptide biomarkers, a comparison between RCAD cases and healthy controls was performed. Only peptides that were detected in at least 70% (frequency threshold) of the samples in at least one of the two groups were further considered for statistical analysis. Using the Wilcoxon rank-sum test followed by adjustment for multiple testing with the false-discovery rate method presented by Benjamini and Hochberg^29^, adjusted P-values were calculated based on the comparison between RCAD cases and healthy controls. Only peptides with a P-value less than 0.05 were considered as statistically significant.

The RCAD146 classifier is developed as SVM classification model^68,69^, based on the amplitudes of the significant urinary peptides related to RCAD, which allows the calculation of specific classification scores. These classification scores were further used for statistical analysis, e.g. ROC curves. In more detail, the sensitivity and specificity assessed for the RCAD146 classifier were calculated based on the number of correctly classified subjects. The receiver operating characteristic (ROC) plots and the confidence intervals (95% CI) were based on exact binomial calculations. The area under the curve (AUC), and sensitivity and specificity values of the ROC of the classifier were determined using R-based statistical software (version 3.3.3) and confirmed using MedCalc version 12.7.5.0 (MedCalc Software bvba, Ostend, Belgium). Graphs related to ROC curves and Box-and-Whisker plot were generated with R-based statistic software (packages ggplot2, plotly).

## Supplementary information

Detailed clinical data from the discovery and validation cohort

Detailed information on the 146 RCAD-related peptides

Proteomic raw CE-MS data

## Data Availability

The raw data generated during and/or analyzed during the current study are available from the corresponding author on reasonable request.
